# Quality of Life in Patients Undergoing Percutaneous Coronary Intervention

**DOI:** 10.3390/clinpract13030057

**Published:** 2023-05-17

**Authors:** Vasiliki Tsoulou, Georgios Vasilopoulos, Theodore Kapadochos, Niki Pavlatou, Antonia Kalogianni, Georgia Toulia, Evangellos Dousis, George Panoutsopoulos, Michael Kourakos, Maria Polikandrioti

**Affiliations:** 1 Department of Nursing, University of West Attica, 12243 Athens, Greece; gvasilop@uniwa.gr (G.V.);; 2 Department of Nutritional Science and Dietetics, University of Peloponnese, 24100 Kalamata, Greece; 3 Department of Nursing, University of Ioannina, 45110 Ioannina, Greece

**Keywords:** percutaneous coronary intervention, quality of life, determinants

## Abstract

Introduction: Percutaneous coronary intervention (PCI) is a non-surgical invasive procedure to treat coronary artery occlusion. The quality of life (QoL) is a way to measure the impact of illness and additionally its treatments to traditional measures of clinical outcomes. Purpose: The aim of the present study was to explore the levels of QoL pre-PCI, 6 and 12 months after PCI, as well as the factors associated with the QoL pre-PCI. Methods: In the present study, 100 patients undergoing PCI were enrolled. Data were collected through the completion of the SF-36 Health Survey (SF-36), which included participants’ characteristics. The statistical significance level was *p* < 0.05. Results: Patients had moderate levels of QoL at baseline, with a median general health score of 45 (IQR: 30–65). A gradual statistically significant increase in scores was observed in all subcategories of the patients’ QoL at 6 and 12 months after PCI (*p* < 0.001). A greater increase in scores was observed in physical functioning, physical role, emotional role and social functionality. In terms of the pre-PCI phase, it was found that physical functionality was statistically significantly associated with educational level (*p* = 0.005), occupation (*p* = 0.026) and whether the patients had children (*p* = 0.041). The physical and emotional role was significantly associated with gender (*p* = 0.046 and *p* = 0.040) and educational level (*p* = 0.030 and *p* = 0.001). Energy–fatigue was significantly associated with gender (*p* = 0.001), age (*p* = 0.028), marital status (*p* = 0.001), educational level (*p* = 0.001), whether the patients had children (*p*= 0.012) and other diseases (*p* = 0.001). Emotional well-being was significantly associated with family history of coronary artery disease (*p* = 0.011) and the frequency of physical exercise (*p* = 0.001). Social functioning was significantly associated with gender (*p* = 0.033), marital status (*p* = 0.034) and educational level (*p* = 0.002). Pain was not found to be significantly associated with patients’ demographics. General health was significantly associated with gender (*p* = 0.003), age (*p* = 0.043), educational level (*p* = 0.001), other diseases (*p* = 0.005) and the frequency of physical exercise (*p* = 0.001). Conclusion: Information about the QoL of PCI and its determinants is important to define an effective and comprehensive care plan.

## 1. Introduction

Coronary artery disease (CAD) is the leading cause of death worldwide, responsible for approximately 16.6% of total deaths in 2016 [1]. Ever since it was first performed in 1977, percutaneous coronary intervention (PCI) has evolved into a widely performed medical procedure in the setting of acute and chronic coronary syndromes [2,3]. During recent years, PCI has advanced at a tremendous pace with the rapid development of new technologies [4] and techniques, including the introduction of drug-eluting stents which lower the chances of restenosis [5].

Annually, PCI is a treatment modality for more than 1 million in the United States [2]. In China, more than 300,000 procedures were performed in 2011, an 18-fold increase compared to 2001 [6], while in India, PCIs are growing at the rate of 14% [5]. In Denmark, approximately 9000 patients are treated annually with PCI, of whom 2500 are less than 65 years old [7]. In Greece, PCI is the preferable treatment option for patients with ST-elevation myocardial infarction in PCI hospitals [8]. However, there are several geographic variations in terms of the organization, operation, management, sustainability and utilization of data collected for PCI registries [1].

The main indication for PCI is angina relief and improvement in the quality of life (QoL) [9]. Interestingly, QoL has become an important outcome measure, since most medical treatments are currently not evaluated only in terms of clinical or biomarker benefits. From a clinical perspective, the measurement of QoL provides essential information to health professionals when planning patient-centered practices. Moreover, QoL is monitoring the performance of clinical care, improving safety and outcomes, thus contributing to treatment cost reduction [10,11]. Furthermore, QoL provides the basis for comparing different treatment options, such as the choice of vascular access site and determining predictors of health benefits [12].

From a practical perspective, data exploring QoL in the field of PCI are useful in developing cost-effective strategies or self-care educational programs to maintain optimal benefits of this minimally invasive procedure. QoL measurements may help health care professionals to motivate beneficial changes in patients’ lifestyles or health behaviors.

Therefore, the aim of this study was to explore QoL levels pre-PCI, 6 and 12 months after PCI, as well as the factors associated with QoL in the pre-PCI phase.

## 2. Materials and Methods

### 2.1. Design, Setting and Period of the Study

In this cross sectional study, 100 patients (69 men and 31 women) who underwent PCI in a public hospital during the period of 2021 to 2022 were enrolled. Participants were selected using the method of convenience sampling. Of the 110 individuals who were on the initial list, 5 patients did not consent to complete the questionnaire and 5 refused to participate after 6 months. Therefore, the data of 100 individuals were analyzed.

### 2.2. Inclusion and Exclusion Criteria of the Sample

Criteria for patients’ inclusion in the study were as follows: (i) age above 18 years; (ii) PCI with drug-eluding stents; (iii) ability to write, read and understand the Greek language; and (iv) ability to read and sign the informed consent form. The exclusion criteria were patients: (i) with a history of mental illness; (ii) visiting clinics to treat some other co-morbidity; (iii) with cognitive disorders and sight or hearing problems; and (iv) with a restenosis in the period under exploration.

### 2.3. Data Collection and Procedure

Data included three measurements: (a) baseline, period up to 1 week before PCI, (b) 6 months after PCI, and (c) 12 months after PCI. The collection of data was performed using the method of interviewing to complete the present research instrument which was specially designed for the purposes of the study. Patients that agreed to participate in the study were invited to a private office room to guarantee their privacy. The QoL measurements at 6 and 12 months were conducted by interview at the hospital when patients had completed their scheduled follow-up. The process of filling out the research instrument lasted approximately between 20 and 30 min.

### 2.4. Research Instrument

The research instrument included patients’ characteristics and QoL assessment using the scale “SF-36 Health Survey (SF-36)”.

Regarding patients’ demographic, the following characteristics were recorded: gender, age, marital status, educational level, occupation, residency and number of children. In terms of clinical characteristics, the following were recorded: type of PCI, family history of CAD, comorbidities, body mass index (BMI), fat and sodium intake, frequency of exercise, smoking and drinking alcohol.

The SF-36, created by Ware and colleagues in 1993, assesses physical and mental health. It consists of 36 questions comprising 8 dimensions: physical functioning, role-physical, role-emotional, energy/fatigue, emotional well-being, social functioning, bodily pain and general health. Respondents have the option to answer each question on Likert-type scales. The scores assigned to the questions are summed separately for the questions assessing the 8 dimensions. Higher score values indicate a better QoL [13].

### 2.5. Ethical Considerations

The present study was approved by the Research Committee of the public hospital and according to the ethical standards of the Declaration of Helsinki (1989) of the World Medical Association. Patients who met the entry criteria were informed by the researcher for the purposes of this study. All patients participated in the study after they had given their written consent. Data collection guaranteed anonymity and confidentiality. All subjects had been informed of their rights to refuse or discontinue participation in the study, Data confidentiality and personal data policy were also respected.

### 2.6. Statistical Analysis

Categorical data are presented with absolute and relative (%) frequencies, while continuous data are presented with the mean, standard deviation, median and interquartile range, where appropriate. The normality of quantitative data was tested with the Kolmogorov–Smirnov test and graphically with histograms. Non-parametric Mann–Whitney and Kruskal–Wallis tests were used to test for an association between QoL and patient characteristics. In addition, multiple linear regression was performed to assess the effect of characteristics on patients’ QoL, adjusting for potential confounders. Results are presented as β-regression coefficients and 95% confidence intervals (95% CI). To test for a trend in QoL scores over time (6 and 12 months after PCI), an ANOVA model for repeated measures was applied, checking for statistically significant effects in the interaction between time and patient characteristics. The observed significance level of 5% was considered statistically significant. All statistical analyses were performed with SPSS version 26 (SPSSInc, Chicago, IL, USA).

## 3. Results

### 3.1. Sample Description

From Table 1, we can observe that 69% of the patients in the sample were men, 64% were over 60 years old, 66% were married and 39% had primary-level education, 55% were retired, 68% lived in Attica and 59% had two children. In addition, 47% underwent facilitated PCI, 84% had hand access, 64% had another family member with coronary artery disease and 66% had some other disease. The mean body mass index (BMI) of the participants was 29.1, while 64% and 60% of patients followed a diet rich in fat and sodium, respectively. In terms of participants’ habits, 53% smoked, 19% consumed alcohol and 88% consumed caffeine, while 47% had no physical exercise at all.

#### 3.1.1. QoL Measurements

Table 2 presents the descriptive measures for patients’ QoL at the three assessment times, pre-PCI, 6 and 12 months later.

Regarding the initial measurement, given the range of values that the scales have (0–100), patients had moderate levels, as the mean values on all subscales were close to 50. A marginally higher QoL was recorded on the subscale “physical pain” (mean value: 53.2 and median 51.3), while the lowest QoL was recorded on the subscale “physical role” (mean value: 23.8 and median 0.0). General health scored a median of 45 (IQR: 30–65). All scales recorded a statistically significant increase in scores 6 and 12 months after PCI (*p* < 0.001). A greater increase in scores was observed in physical functioning, physical role, emotional role and social functioning.

#### 3.1.2. Association between QoL and Patient Characteristics

Table 3, Table 4 and Table 5 present the patient characteristics that are significantly associated with QoL before PCI.

Regarding physical functioning (Table 3), it was found to be significantly associated with the level of education (*p* = 0.005), occupation (*p* = 0.026), number of children (*p* = 0.041) and frequency of physical exercise (*p* = 0.001). More specifically, patients with a primary education had worse physical functioning (median 10) than those with a secondary education (median 45) and those with a university education (median 50). Retired patients had worse physical functioning (median 25) than employees (median 50). Furthermore, the more children the patients had, the worse the physical functioning. Those who never exercised (median 10) had correspondingly worse physical functioning than those who exercised 1–2 times/week (median 45) and those who exercised >2 times/week (median 82.5).

Regarding the physical role (Table 3), it was found to be significantly associated with gender (*p* = 0.046), education level (*p* = 0.030) and frequency of physical exercise (*p* = 0.026). More specifically, male patients had a better physical role (median 12.5) than female patients (median 0). Similarly, patients with a higher education had a better physical role (median 12.5) than patients with a lower level of education (median 0). Furthermore, patients who exercised >2 times/week had a better physical role (median 25) than those who exercised 1–2 times/week or never (median 0).

Regarding the emotional role (Table 3), it was found to be significantly associated with gender (*p* = 0.040), education level (*p* = 0.001), occupation (*p* = 0.032) and current smoking (*p* = 0.020). More specifically, male patients had a better emotional role (median 33.3) than female patients (median 0). Similarly, patients with university education had a better emotional role (median 100) than those with a secondary education (median 66.7) and those with a primary education (median 0). Patients who worked had a better emotional role (median 100) than retired patients (median 0). In addition, patients who smoked had a better emotional role (median 83.3) than nonsmokers (median 0).

Regarding energy–fatigue (Table 4), it was found to be significantly associated with gender (*p* = 0.001), age (*p* = 0.028), marital status (*p* = 0.001), education level (*p* = 0.001), occupation (*p* = 0.001), number of children (*p* = 0.012), whether they had other diseases (*p* = 0.001) and the frequency of physical exercise (*p* = 0.001). More specifically, female patients had worse energy–fatigue (median 35) than men (median 55). Patients older than 70 years had worse energy–fatigue (median 35) than younger patients (median 55). Widowed patients also had worse energy–fatigue (median 25) than married (median 52.5) and single patients (median 60). Patients with a primary education had worse energy–fatigue (median 30) than those with a secondary education (median 55) and those with a university education (median 60). Retired patients and those with some other disease had worse energy–fatigue (median 35 and 40, respectively) than workers and those without another disease (median 60). Furthermore, the more children the patients had, the worse the energy–fatigue. Those who never exercised (median 35) had correspondingly worse energy–fatigue than those who exercised 1–2 times/week (median 55) and those who exercised >2 times/week (median 70).

Regarding emotional well-being (Table 4), it was found to be significantly associated with family history of coronary artery disease (*p* = 0.011) and the frequency of physical exercise (*p* = 0.001). More specifically, patients with a family history of coronary artery disease had worse emotional well-being (median 40) than those without a family history (median 52). In addition, patients who exercised >2 times/week had better emotional well-being (median 56) than those who exercised 1–2 times/week (median 48) or never (median 40).

Regarding social functioning (Table 4), it was found to be significantly associated with gender (*p* = 0.033), marital status (*p* = 0.034), education level (*p* = 0.002) and occupation (*p* = 0.018). More specifically, male patients had better social functioning (median 50) than female patients (median 37.5). In contrast, widowed patients had worse social functioning (median 31.3) than married and single patients (median 50). Patients with a primary education had worse social functioning (median 37.5) than those with a secondary education (median 50) and those with a university education (median 62.5).

Regarding physical pain (Table 5), it was not found to be significantly associated with any of the patient characteristics.

Regarding general health (Table 5), it was found to be statistically significantly associated with gender (*p* = 0.003), age (*p* = 0.043), education level (*p* = 0.001), occupation (*p* = 0.001), whether they had some other disease (*p* = 0.005) and the frequency of physical exercise (*p* = 0.001). More specifically, female patients had worse general health (median 35) than male patients (median 50). Patients over 60 had worse general health (median 40) than younger patients (median 55). Patients with a primary education had worse general health (median 35) than those with a secondary education (median 50) and those with a university education (median 62.5). Retired patients and those with some other disease had worse general health (median 40) than workers and those without some other disease (median 60 and 62.5, respectively). Those who never exercised (median 35) had correspondingly worse general health than those who exercised 1–2 times/week (median 50) and those who exercised >2 times/week (median 65).

#### 3.1.3. Effect of Patient Characteristics on QoL at Baseline

Multiple linear regression was performed with the individual sub-scales of QoL as the dependent variables to infer which of the patient characteristics (playing the role of independent factors) remained statistically significant, and thus had an impact on the quality of life, while correcting for potential confounding factors.

From Table 6, we can conclude that patients who exercised >2 times/week had 34.6 point-better physical functionality compared to those who never exercised (β = 34.6, (95% CI: 13.2–56.0), *p* = 0.001). Regarding the physical role, it can also be observed that patients who exercised >2 times/week had a 19.2 point-better physical role compared to those who never exercised (β = 19.2, (95% CI: 2.4–40.8), *p* = 0.041). Regarding the emotional role, it can be observed that patients with a secondary and university education had a better emotional role by 28.5 and 51.2 points, respectively, compared to patients with a primary education (β = 28.5, 95% CI: (5.8–51.3), *p* = 0.015 and β = 51.2, 95% CI:(23.4–78.9), *p* = 0.001, respectively). Similarly, patients who smoked had a better emotional role by 20.3 points than patients who did not smoke (β = 20.3, 95% CI: (1.9–38.8), *p* = 0.031). Regarding energy–fatigue, patients aged 60–70 years had 14.8-point better energy–fatigue than patients aged <60 years (β = 14.8, (95% CI: 2.9–26.5), *p* = 0.015). Patients with a secondary and university education had better energy–fatigue by 13.2 and 12.2 units, respectively, than patients with a primary education (β = 13.2, 95% CI: (3.3–23.2), *p*= 0.010 and β = 12.2, 95% CI: (0.4–24.8), *p* = 0.048, respectively). Similarly, patients who exercised >2 times/week had 16.4-point better energy–fatigue than those who never exercised (β = 16.4, (95% CI: 5.7–27.1), *p* = 0.003). Regarding emotional well-being, it can also be observed that patients who had a family history of coronary artery disease had 7.8-point worse mental health compared to patients who did not (β = −7.8, 95% CI: (−14.8–0.7), *p* = 0.031). In addition, patients who exercised >2 times/week had 14.8-point better emotional well-being than those who never exercised (β = 14.8, (95 % CI: 5.7–23.7), *p* = 0.002). In terms of social functioning, it can be observed that patients with a higher education had better social functioning by 12.8 units compared to patients with a primary education (β= 12.8, 95% CI: (0.7–26.4), *p* = 0.044). Finally, regarding general health, those with a secondary and university education had better general health by 17.2 and 20.9 points, respectively, compared to patients with a primary education (β = 17.2, 95% CI: (7.7–26.6), *p* = 0.001 and β = 20.9, 95% CI: (8.9–32.8), *p* = 0.001, respectively). Similarly, patients who exercised >2 times/week had 14.7 units of better general health compared to those who never exercised (β = 14.7, (95% CI: 4.5–24.8), *p*= 0.005).

#### 3.1.4. Effects of Patient Characteristics on QoL Trends

Through repeated measures modeling, it was assessed whether there was a statistically significant interaction between patient characteristics and recording time (thus significant differences in the trend of the subscale scores).

Regarding physical functioning, a statistically significant interaction was observed between the time and frequency of physical exercise (*p* = 0.001). More specifically, patients who never exercised or exercised 1–2 times/week had a greater increase in the physical functioning score over time compared to patients who exercised >2 times/week, where a stability of scores over time was observed. In regard to the physical role, a statistically significant interaction was observed between time and age (*p* = 0.018), as well as family history of coronary artery disease (*p* = 0.015). More specifically, patients >70 years old had a lower tendency to increase the physical role score over time compared to patients <60 years old and 60–70 years old, in whom a greater tendency to increase the score was observed. Accordingly, patients with a family history of coronary artery disease had a greater tendency to increase the score compared to patients without a history. Regarding the emotional role, no statistically significant interaction was observed between time and patient characteristics. In terms of energy–fatigue, no statistically significant interaction between time and patient characteristics was observed. In regard to emotional well-being, a statistically significant interaction was observed between time and age (*p* = 0.011), as well as family history of coronary artery disease (*p* = 0.004). More specifically, patients >70 years old had a lower tendency to increase the score over time compared to patients <60 years old and 60–70 years old, in whom a greater tendency to increase the score was observed. Accordingly, patients with a family history of coronary artery disease had a greater tendency to increase the score compared to patients without a history. Regarding social functioning, no statistically significant interaction between time and patient characteristics was observed. In terms of physical pain, a statistically significant interaction was observed between time and age (*p* = 0.004), as well as family history of coronary artery disease (*p* = 0.003). More specifically, patients >70 years old had a lower tendency to increase the score of physical pain over time compared to patients <60 years old and 60–70 years old, in whom a greater tendency to increase the score was observed. In fact, patients >70 years old had a lower physical pain score 12 months after PCI compared to the recording of the previous six months (6 months after PCI). Accordingly, patients with a family history of coronary artery disease had a greater tendency to increase the score compared to patients without a history. Regarding general health, a statistically significant interaction was observed between time and age (*p* = 0.006), as well as family history of coronary artery disease (*p* = 0.012). More specifically, patients >70 years old tended to decrease the general health score over time, while patients <60 years old and 60–70 years old showed a tendency to increase the score. Accordingly, patients with a family history of coronary artery disease tended to increase the score, while in patients without a history, a stability of the scores was observed.

## 4. Discussion

The results of the present study showed moderate levels of QoL in the pre-PCI period, and an increase in QoL scores 6 and 12 months post-PCI. The QoL measurement prior to this minimally invasive procedure provides significant insights into the selection of patients and offers a base to clinicians to provide individualized care afterward [6,12,14].

In the pre-PCI period, regarding gender, a lower QoL was observed in women across all subscales, apart from physical functioning, emotional well-being and physical pain. This finding is similar to other relevant studies conducted worldwide. For example, in Australia, among 16,517 patients (22.9% women), the female sex was a predictor of a poor QoL after PCI for acute coronary syndromes (ACSs), including anxiety and depression [15]. Contrariwise, in Poland no significant differences in the QoL between the sexes were found in a 36-month follow-up of PCI [16]. In Netherlands, after coronary revascularization (coronary artery bypass graft or PCI), women reported a slow improvement in physical state, irrespective of the comorbidity burden [17]. In a period of 12 months post-PCI, women in Vietnam showed a better recovery in mobility, despite having had a worse QoL 30 days after discharge [18], whereas those in the “Antiplatelet Therapy Observational Registry” reported a lower QoL [19]. Clinical factors may possibly affect QoL, as women have a higher prevalence of diabetes mellitus, systemic hypertension, chronic renal insufficiency, peripheral arterial disease, congestive heart failure, as well as a lower body surface area and higher body mass index [20]. Furthermore, women may invest their experiences with a different personal meaning. Perceptions affect treatment and expectations or efforts for recovery, as well as participation in rehabilitation programs [21]. Beyond the shadow of doubt, all the aforementioned parameters influence the QoL.

As far as age is concerned in the pre-PCI phase, participants over 70 years old and those 61–70 years had a worse QoL in the subscale of energy–fatigue and general health, respectively. In general terms, elderly patients have a better QoL compared to: (i) the pre-PCI phase, (ii) patients who follow conservative treatment, (iii) age-matched general population and an equivalent or superior QoL compared to younger patients who underwent PCI. These benefits are observed for at least one year [22], while the greatest improvement is noticed in physical health compared to young groups [23]. Possibly, the elderly derive greater benefits from revascularization, as they have more cardiovascular risk factors and a greater burden of ischemic disease. Another aspect that could explain the better health status in the elderly is that they are more accepting of their functional impairment. Noteworthy, the elderly are more likely to experience procedural complications owing to age-related physiological changes, frailty, or comorbidities, and are less likely to be employed or have dependents requiring their support [23,24]. Despite the risks of performing PCI in elderly patients, the decision must be considered in relation to benefits for QoL. Peri-procedural mortality rates appear to be higher in the elderly, but if they survive this procedure, they lead an acceptable QoL. Given these benefits, it is important not to abandon elderly patients in the inadequate management of conservative treatment. Delay or exclusion from intervention or research studies solely based on age may reject patients from receiving the best evidenced practice care [22]. Finally, and most strikingly, age per se should not deter against revascularization, since there are QoL benefits [23].

Moreover, in the pre-PCI phase, participants with a primary education had worse physical and social functioning, emotional and physical roles, energy–fatigue and general health. A possible explanation is that education supports patients to develop adaptive mechanisms and become able to handle their health needs more effectively. A low level of education seems to affect the QoL through reduced use of preventive health services, less awareness of their medical condition and poor self-care behaviors [25]. Additionally, the level of education either hinders or promotes the understanding of information provided by health professionals [26]. Education changes people’s attitude and leads to improvements in the QoL [27]. Patients with a higher income and education experienced a better QoL 6 months after revascularization [28].

According to the present results, in the pre-PCI phase, the retired participants had a worse QoL in the domains of physical and social functioning, emotional role, energy–fatigue and general health. Participants who still work possibly keep in contact with other individuals, maintain communication and receive social support, thus enjoying a better QoL [29]. However, in developing countries, the low socio-economic status (income, occupation, education) is associated with a higher incidence of major adverse cardiac events post-PCI, thus indirectly influencing the QoL. More specifically, patients with a low socio-economic status are less adherent to medication and therapeutic advice after PCI. At the 12-month follow-up, the revascularization repeat and the recurrent myocardial infraction were higher in the low socio-economic group [30]. Therefore, evaluating the socio-economic status in the pre-PCI period is essential to take the necessary steps post-PCI.

Widowed patients had a worse QoL in energy–fatigue and social functioning in the pre-PCI phase. This finding is partially attributed to diminished support compared to those living in marital bonds. The prevailing view is that family support is associated with health-promoting self-management and adherence to treatment. Moreover, social interaction promotes health because it maintains a rhythm of life [31,32,33]. It is likely that family support provides a sense of security and a peaceful environment to individuals, which enhances their confidence to overcome disease-related difficulties, thus improving QoL. [10,11] An increase in social support by significant ones, family, or friends leads to a decrease in state and trait anxiety among cardiac patients [32]. Apart from the lack of supportive environment, the widowed may experience difficulties in handling practical issues or performing daily activities alone. Therefore, a trusting relationship with health professionals may improve the QoL by presenting treatment options with clarity and actively enhancing participation in the decision-making process [29,34].

Moreover, patients who did not smoke had a worse QoL in the emotional role subscale. QoL evidence promotes smokers and health professionals to become more sensitive about the adverse effects of smoking. Notably, smoking increases the risk of myocardial infarction and death in patients with heart disease, especially after PCI. Smoking limits vascular reconstruction and coronary blood flow by creating microvascular endothelial dysfunction, and reduces the ability to exercise. Thus, smoking may diminish the QoL [27]. Moreover, patients with a family history of coronary artery disease had a worse QoL in the dimension of emotional well-being. Contrariwise, evidence supports that individuals having a positive cardiac family history may better comprehend the important role of self-care, thus improving the QoL [27].

Patients who never exercised had a worse QoL in physical functioning, physical role, energy–fatigue, emotional well-being and general health. It is widely known that the treatment of coronary artery disease involves interventions (diet, risk factors modification, exercise) beyond pharmacologic therapy and coronary revascularization. Exercise plays a vital role in the QoL improvement. A 12-week exercise cardiac rehabilitation showed greater improvements in maximal oxygen uptake among elderly patients undergoing PCI [35]. Likewise, an improvement in health status after PCI for chronic total occlusion was associated with participation in regular exercise [36]. Early home-based exercise in patients with myocardial infraction who underwent PCI may improve cardiac function, reduce postoperative complications, and enhance cardiac antioxidant capacity and exercise ability, thus promoting the QoL [37]. A cardiac rehabilitation program using home exercise training with wireless monitoring led to the improvement of both exercise capacity and QoL in patients undergoing PCI [38]. Developing interventions to safely increase exercise in this vulnerable population may improve the QoL.

Last but not least, shaping future and appropriate interventions demands an in depth understanding of patients’ perceptions associated with QoL in the pre-PCI period. Unfortunately, some patients underestimate cardiac disease for various reasons, such as the short time of procedure and hospital stay, prompt improvement of symptoms and early return to prior activities [39]. In Sweden, among 1073 patients after PCI, 67% perceived that they were cured, 38% declared no need to change their habits, 16% continued to use tobacco and fewer than 50% were regularly physically active. Nutritional counseling was provided to 71%, but only 40% changed food habits. Only 27% reported that they still had cardiovascular disease and needed behavioral change [40]. If in such cases is added the psychological stress in cardiac illness, then treatment becomes more complicated [41].

The results of the present study showed an increase in the QoL score 6 and 12 months post-PCI, with a greater improvement in physical functioning, physical role, emotional role and social functioning. Similarly, in the United Kingdom, PCI improved the QoL, especially physical functioning, vitality and general health, at both 3 months and 1 year, but not at three years [14].

The following results were observed at 6- and 12-month QoL measurements: (i) patients who never exercised or exercised 1–2 times/week had a greater increase in physical functioning score, (ii) patients >70 years old had a lower tendency to increase the score in physical role, emotional well-being, social functioning, physical pain and (iii) patients with a family history of coronary disease tended to increase the score in physical role, emotional well-being, physical pain and general health. Among participants that were 61–70 years old, a great tendency to increase the score of physical role, emotional well-being, social functioning, physical pain and general health was observed.

At the 3-year follow-up after PCI, the significant independent determinants of a lower QoL included the female sex, age >60 years and diabetes mellitus [16]. According to van den Berge et al., [42] studies including a 12-month follow-up have shown that age, the male gender, renal impairment, smoking and prior coronary artery bypass grafting were predictors of health status post-PCI. At 10 years post-PCI, the SF-36 scores at baseline, age and previous PCI were significant predictors of subjective health status. Evaluating the QoL at baseline is a useful indicator to predict the long-term subjective health status [42]. Modification of the SF-36 score is a key challenge for clinicians involved in the care of PCI.

## 5. Limitations of the Study

Limitations of this study include the cross-sectional design which is not allowing evidence of causal relationship. Convenience sampling in a single-center study in Attica is one more limitation as this method is not representative of all PCI patients living in Greece, thus limiting the generalizability of the results. The sample size might be a small one, though most statisticians agree that the minimum sample size to obtain meaningful results is 100. Furthermore, patients were selected in regard to their type of stent (drug-eluting stent, DES). Finally, were not explored other parameters that are shown to influence the QoL, such as cardiac rehabilitation, mental health status and psychosocial support.

## 6. Conclusions

The present study showed a moderate QoL pre-PCI and an improvement 6 and 12 months afterward. Over time after PCI, patients aged >70 years had a lower tendency to increase the QoL score, whereas patients with a family history of coronary disease tended to increase the QoL. Moreover, a greater increase in physical functioning score over time was observed in patients who never exercised or exercised 1–2 times/week.

Prior to PCI, a worse QoL was observed: (i) in patients with a primary education, more than one child, who were retired and never exercised in regards to physical functioning; (ii) in female patients, patients with a primary education and those who never exercised in regards to the physical role; (iii) in female patients, those with a primary education, retired ones and those who did not smoke in regards to the emotional role; (iv) in female patients, the widowed, the retired ones, those older than 70 years, as well as those with a primary education, with some other disease, more than one child and those who never exercised in regards to energy–fatigue; (v) in patients with a family history of coronary artery disease and those who never exercised in regards to emotional well-being; (vi) in female patients, widowed ones, those with a primary education and retired ones in regards to social functioning; and (vii) in female patients, over 60 years old, with a primary education, retired ones, with some other disease and those who never exercised in regards to general health.

Further research should explore the determinants of the QoL in larger multicenter studies. Needless to say, the QoL is an undeniable right in any society.

## Figures and Tables

**Table 1:** Distribution of the sample (*n* = 100).

	*n* (%)		*n* (%)
Demographics			
Gender (Men)	69 (69.0%)	Occupation	
Age (years)		Uemployed	1 (1.0%)
30–40	1 (1.0%)	Public employee	6 (6.0%)
41–50	11 (11.0%)	Private employee	10 (10.0%)
51–60	24 (24.0%)	Freelancer	16 (16.0%)
61–70	31 (31.0%)	Household	9 (9.0%)
71–80	33 (33.0%)	Pensioner	55 (55.0%)
Family Status		Other	3 (3.0%)
Married	66 (66.0%)	Residency	
Single	6 (6.0%)	Attica	68 (68.0%)
Divorced	8 (8.0%)	Country Capital	15 (15.0%)
Widowed	18 (18.0%)	Small Town	5 (5.0%)
Living Together	2 (2.0%)	Village	12 (12.0%)
Education Level		No of Children	
Primary	39 (39.0%)	None	12 (12.0%)
Secondary	39 (39.0%)	One	14 (14.0%)
University	20 (20.0%)	Two	59 (59.0%)
MSc-PhD	2 (2.0%)	More than 2	15 (15.0%)
Clinical characteristics and habits			
Type		Smoking (Yes)	53 (53.0%)
Primary	36 (36.0%)	Alcohol	
Rescue	17 (17.0%)	No	46 (46.0%)
Facilitated	47 (47.0%)	Yes	19 (19.0%)
CAD-Family history (Yes)	64 (64.0%)	Occasionally	35 (35.0%)
Other Disease (Yes)	66 (66.0%)	Exercise	
ΒΜΙ	29.1 (4.8) *	Never	47 (47.0%)
Diet rich in fat		1–2 times/week	33 (33.0%)
No	13 (13.0%)	>2 times/week	20 (20.0%)
Yes	64 (64.0%)		
Occasionally	23 (23.0%)		
Diet rich in sodium			
No	15 (15.0%)		
Yes	60 (60.0%)		
Occasionally	25 (25,0%)		

* mean (standard deviation).

**Table 2:** QoL scores.

	Baseline		6 Months after PCI		12 Months after PCI		
	Mean (SD)	Median (IQR)	Mean (SD)	Median (IQR)	Mean (SD)	Median (IQR)	*p*-Value *
Physical Functioning	42.6 (37.8) ^¥^	47.5 (0.0–77.5)	71.4 (22.9)	75.0 (50.0–90.0)	84.1 (21.5) ^£^	90.0 (80.0–100.0)	<0.001
Physical Role	23.8 (39.3) ^¥^	0.0 (0.0–50.0)	45.5 (45.8)	25.0 (0.0–100.0)	57.8 (49.4) ^£^	100.0 (0.0–100.0)	<0.001
Emotional Role	45.1 (46.7) ^¥^	33.3 (0.0–100.0)	55.0 (44.0)	66.7 (0.0–100.0)	60.8 (48.1) ^£^	100.0 (0.0–100.0)	<0.001
Energy–Fatigue	47.6 (22.5) ^¥^	50.0 (30.0–65.0)	52.8 (22.6)	55.0 (35.0–70.0)	56.5 (22.0)	60.0 (40.0–70.0)	<0.001
Emotional Well-Being	47.9 (18.4) ^¥^	46.0 (36.0–58.0)	55.7 (16.0)	56.0 (44.0–68.0)	62.4 (17.5) ^£^	64.0 (52.0–76.0)	<0.001
Social Functioning	47.8 (21.7) ^¥^	50.0 (25.0–62.5)	69.0 (22.2)	62.5 (50.0–87.5)	71.5 (22.3)	75.0 (50.0–87.5)	<0.001
Pain	53.2 (27.7) ^¥^	51.3 (32.5–76.3)	73.0 (22.0)	77.5 (57.5–90.0)	71.3 (25.2)	77.5 (45.0–100.0)	<0.001
General Health	46.9 (20.7) ^¥^	45.0 (30.0–65.0)	49.9 (19.3)	45.0 (35.0–65.0)	54.3 (23.9) ^£^	50.0 (35.0–75.0)	<0.001

SD: standard deviation, IQR: interquartile range * *p*-value assessed by repeated measures ANOVA ^¥^ Statistically significant different score from the other two time points after multiple comparisons. ^£^ Statistically significant different score from the 6 m time point after multiple comparisons.

**Table 3:** Association of patients’ characteristics with baseline QoL (*n* = 100).

	Physical Functioning		Physical Role		Emotional Role	
	Median (IQR)	*p*-Value	Median (IQR)	*p*-Value	Median (IQR)	*p*-Value
Gender		0.066		0.046		0.040
Male	50.0 (0.0–85.0)		12.5 (0.0–75.0)		33.3 (0.0–100.0)	
Female	30.0 (0.0–50.0)		0.0 (0.0–0.0)		0.0 (0.0–66.7)	
Age (years)		0.228		0.555		0.098
≤60	50.0 (7.5–92.5)		0.0 (0.0–75.0)		66.7 (0.0–100.0)	
61–70	40.0 (0.0–70.0)		0.0 (0.0–25.0)		50.0 (0.0–100.0)	
>70	30.0 (0.0–50.0)		0.0 (0.0–50.0)		0.0 (0.0–66.7)	
Family Status		0.943		0.139		0.053
Married/Living Together	42.5 (0.0–75.0)		0.0 (0.0–37.5)		33.3 (0.0–100.0)	
Single/Divorced	40.0 (0.0–95.0)		12.5 (0.0–100.0)		66.7 (0.0–100.0)	
Widowed	50.0 (0.0–55.0)		0.0 (0.0–0.0)		0.0 (0.0–33.3)	
Education Level		0.005		0.030		0.001
Primary	10.0 (0.0–50.0)		0.0 (0.0–0.0)		0.0 (0.0–33.3)	
Secondary	45.0 (0.0–85.0)		0.0 (0.0–75.0)		66.7 (0.0–100.0)	
University	50.0 (40.0–95.0)		12.5 (0.0–75.0)		100.0 (66.7–100.0)	
Occupation		0.026		0.105		0.032
Employee	50.0 (15.0–95.0)		0.0 (0.0–75.0)		100.0 (0.0–100.0)	
Pensioner	25.0 (0.0–55.0)		0.0 (0.0–0.0)		0.0 (0.0–100.0)	
Residency		0.308		0.579		0.246
Attica	50.0 (0.0–85.0)		0.0 (0.0–75.0)		33.3 (0.0–100.0)	
County Capital	25.0 (0.0–50.0)		0.0 (0.0–0.0)		0.0 (0.0–100.0)	
Small Town/Village	30.0 (0.0–50.0)		0.0 (0.0–50.0)		0.0 (0.0–100.0)	
No. of Children		0.041		0.053		0.148
None	95.0 (7.5–100.0)		75.0 (0.0–100.0)		100.0 (33.3–100.0)	
One	50.0 (10.0–85.0)		0.0 (0.0–75.0)		33.3 (0.0–100.0)	
Two	40.0 (0.0–65.0)		0.0 (0.0–25.0)		0.0 (0.0–100.0)	
More than 2	0.0 (0.0–50.0)		0.0 (0.0–0.0)		0.0 (0.0–66.7)	
PCI Type		0.156		0.157		0.411
Primary	50.0 (2.5–85.0)		0.0 (0.0–25.0)		66.7 (0.0–100.0)	
Rescue	20.0 (0.0–50.0)		0.0 (0.0–0.0)		0.0 (0.0–100.0)	
Facilitated	50.0 (0.0–85.0)		0.0 (0.0–75.0)		33.3 (0.0–100.0)	
CAD Family History		0.117		0.123		0.342
No	50.0 (15.0–92.5)		0.0 (0.0–87.5)		50.0 (0.0–100.0)	
Yes	30.0 (0.0–72.5)		0.0 (0.0–12.5)		0.0 (0.0–100.0)	
Other Disease		0.144		0.294		0.199
No	57.5 (0.0–85.0)		0.0 (0.0–75.0)		83.3 (0.0–100.0)	
Yes	32.5 (0.0–55.0)		0.0 (0.0–25.0)		0.0 (0.0–100.0)	
Diet Rich in Fat		0.719		0.652		0.526
No	20.0 (0.0–90.0)		0.0 (0.0–0.0)		33.3 (0.0–100.0)	
Yes/Occasionally	50.0 (0.0–75.0)		0.0 (0.0–50.0)		33.3 (0.0–100.0)	
Diet Rich in Sodium		0.712		0.207		0.339
No	20.0 (0.0–100.0)		0.0 (0.0–100.0)		100.0 (0.0–100.0)	
Yes/Occasionally	50.0 (0.0–65.0)		0.0 (0.0–25.0)		33.3 (0.0–100.0)	
Smoking		0.550		0.952		0.020
No	40.0 (0.0–80.0)		0.0 (0.0–75.0)		0.0 (0.0–100.0)	
Yes	50.0 (0.0–75.0)		0.0 (0.0–50.0)		83.3 (0.0–100.0)	
Alcohol		0.770		0.999		0.052
No	50.0 (0.0–80.0)		0.0 (0.0–75.0)		0.0 (0.0–100.0)	
Yes/Occasionally	42.5 (5.0–75.0)		0.0 (0.0–25.0)		66.7 (0.0–100.0)	
Exercise		0.001		0.026		0.063
Never	10.0 (0.0–50.0)		0.0 (0.0–0.0)		0.0 (0.0–100.0)	
1–2 times/week	45.0 (5.0–70.0)		0.0 (0.0–0.0)		100.0 (0.0–100.0)	
>2 times/week	82.5 (50.0–100.0)		25.0 (0.0–100.0)		66.7 (0.0–100.0)	

**Table 4:** Association of patients’ characteristics with baseline QoL (*n* = 100).

	Energy–Fatigue		Emotional Well-Being		Social Functioning	
	Median (IQR)	*p*-Value	Median (IQR)	*p*-Value	Median (IQR)	*p*-Value
Gender		0.001		0.053		0.033
Male	55.0 (40.0–70.0)		48.0 (40.0–60.0)		50.0 (37.5–62.5)	
Female	35.0 (15.0–55.0)		40.0 (32.0–52.0)		37.5 (25.0–50.0)	
Age (years)		0.028		0.392		0.353
≤60	55.0 (37.5–70.0)		44.0 (34.0–60.0)		50.0 (37.5–62.5)	
61–70	55.0 (35.0–60.0)		40.0 (36.0–56.0)		50.0 (37.5–50.0)	
>70	35.0 (15.0–60.0)		48.0 (40.0–60.0)		37.5 (25.0–75.0)	
Family Status		0.001		0.275		0.034
Married/Living Together	52.5 (35.0–67.5)		48.0 (36.0–60.0)		50.0 (37.5–62.5)	
Single/Divorced	60.0 (55.0–65.0)		44.0 (32.0–52.0)		50.0 (37.5–75.0)	
Widowed	25.0 (15.0–40.0)		44.0 (28.0–48.0)		31.3 (25.0–50.0)	
Education Level		0.001		0.115		0.002
Primary	30.0 (15.0–50.0)		44.0 (32.0–48.0)		37.5 (25.0–50.0)	
Secondary	55.0 (40.0–65.0)		52.0 (36.0–60.0)		50.0 (37.5–62.5)	
University	60.0 (55.0–70.0)		46.0 (40.0–76.0)		62.5 (50.0–75.0)	
Occupation		0.001		0.425		0.018
Employee	60.0 (50.0–70.0)		48.0 (40.0–60.0)		50.0 (37.5–62.5)	
Pensioner	35.0 (20.0–60.0)		44.0 (32.0–56.0)		37.5 (25.0–62.5)	
Residency		0.598		0.171		0.996
Attica	50.0 (30.0–65.0)		48.0 (38.0–60.0)		43.8 (25.0–62.5)	
County Capital	55.0 (25.0–60.0)		36.0 (32.0–52.0)		50.0 (37.5–50.0)	
Small Town/Village	40.0 (30.0–55.0)		44.0 (40.0–56.0)		50.0 (37.5–62.5)	
No. of Children		0.012		0.299		0.219
None	65.0 (45.0–80.0)		50.0 (40.0–62.0)		50.0 (31.3–81.3)	
One	60.0 (35.0–70.0)		50.0 (32.0–80.0)		50.0 (37.5–75.0)	
Two	50.0 (25.0–65.0)		44.0 (40.0–56.0)		37.5 (25.0–62.5)	
More than 2	35.0 (25.0–50.0)		36.0 (28.0–56.0)		37.5 (25.0–75.0)	
PCI Type		0.185		0.155		0.362
Primary	52.5 (32.5–67.5)		40.0 (34.0–52.0)		50.0 (37.5–62.5)	
Rescue	40.0 (20.0–55.0)		48.0 (32.0–52.0)		50.0 (25.0–50.0)	
Facilitated	55.0 (30.0–65.0)		48.0 (40.0–60.0)		37.5 (25.0–62.5)	
CAD Family History		0.742		0.011		0.290
No	50.0 (35.0–65.0)		52.0 (40.0–64.0)		50.0 (37.5–75.0)	
Yes	50.0 (25.0–65.0)		40.0 (32.0–52.0)		43.8 (25.0–62.5)	
Other Disease		0.001		0.910		0.182
No	60.0 (50.0–70.0)		48.0 (40.0–56.0)		50.0 (37.5–62.5)	
Yes	40.0 (25.0–60.0)		44.0 (36.0–60.0)		37.5 (25.0–62.5)	
Diet Rich in Fat		0.388		0.523		0.677
Nof	40.0 (30.0–55.0)		48.0 (40.0–52.0)		50.0 (25.0–62.5)	
Yes/Occasionally	50.0 (30.0–65.0)		44.0 (36.0–60.0)		50.0 (37.5–62.5)	
Diet Rich in Sodium		0.801		0.110		0.208
No	55.0 (25.0–65.0)		52.0 (40.0–60.0)		50.0 (37.5–75.0)	
Yes/Occasionally	50.0 (30.0–65.0)		44.0 (36.0–56.0)		37.5 (25.0–62.5)	
Smoking		0.500		0.862		0.347
No	45.0 (25.0–65.0)		44.0 (36.0–56.0)		37.5 (25.0–62.5)	
Yes	55.0 (35.0–60.0)		48.0 (36.0–60.0)		50.0 (37.5–62.5)	
Alcohol		0.539		0.827		0.806
No	45.0 (25.0–65.0)		48.0 (32.0–60.0)		43.8 (25.0–62.5)	
Yes/Occasionally	55.0 (35.0–60.0)		40.0 (36.0–56.0)		50.0 (37.5–62.5)	
Exercise		0.001		0.001		0.112
Never	35.0 (20.0–55.0)		40.0 (32.0–52.0)		37.5 (25.0–50.0)	
1–2 times/week	55.0 (35.0–65.0)		48.0 (40.0–60.0)		50.0 (37.5–62.5)	
>2 times/week	70.0 (47.5–80.0)		56.0 (46.0–74.0)		50.0 (37.5–68.8)	

**Table 5:** Association of patients’ characteristics with baseline QoL (*n* = 100).

	Pain		General Health	
	Median (IQR)	*p*-Value	Median (IQR)	*p*-Value
Gender		0.989		0.003
Male	52.5 (22.5–75.0)		50.0 (35.0–70.0)	
Female	50.0 (32.5–77.5)		35.0 (25.0–50.0)	
Age (years)		0.348		0.043
≤60	50.0 (22.5–70.0)		55.0 (42.5–67.5)	
61–70	60.0 (32.5–87.5)		40.0 (30.0–65.0)	
>70	45.0 (32.5–67.5)		40.0 (25.0–50.0)	
Family Status		0.831		0.289
Married/Living Together	53.8 (22.5–77.5)		45.0 (30.0–65.0)	
Single/Divorced	46.3 (25.0–67.5)		52.5 (30.0–65.0)	
Widowed	47.5 (32.5–75.0)		35.0 (25.0–55.0)	
Education Level		0.182		0.001
Primary	42.5 (22.5–67.5)		35.0 (25.0–45.0)	
Secondary	47.5 (32.5–75.0)		50.0 (30.0–65.0)	
University	62.5 (32.5–87.5)		62.5 (50.0–75.0)	
Occupation		0.298		0.001
Employee	57.5 (25.0–77.5)		60.0 (45.0–70.0)	
Pensioner	45.0 (22.5–67.5)		40.0 (25.0–55.0)	
Residency		0.436		0.551
Attica	53.8 (32.5–77.5)		47.5 (32.5–65.0)	
County Capital	45.0 (22.5–55.0)		45.0 (25.0–60.0)	
Small Town/Village	57.5 (32.5–87.5)		35.0 (30.0–55.0)	
No. of Children		0.495		0.054
None	57.5 (46.3–85.0)		55.0 (42.5–72.5)	
One	55.0 (32.5–65.0)		55.0 (30.0–75.0)	
Two	45.0 (22.5–75.0)		45.0 (30.0–60.0)	
More than 2	57.5 (32.5–77.5)		35.0 (25.0–45.0)	
PCI Type		0.564		0.177
Primary	48.8 (22.5–76.3)		50.0 (32.5–65.0)	
Rescue	55.0 (32.5–70.0)		35.0 (25.0–50.0)	
Facilitated	50.0 (32.5–77.5)		45.0 (30.0–65.0)	
CAD Family History		0.086		0.262
No	57.5 (38.8–77.5)		50.0 (35.0–65.0)	
Yes	45.0 (22.5–71.3)		45.0 (25.0–65.0)	
Other Disease		0.100		0.005
No	57.5 (35.0–87.5)		62.5 (40.0–70.0)	
Yes	45.0 (25.0–67.5)		40.0 (30.0–55.0)	
Diet Rich in Fat		0.526		0.423
No	60.0 (32.5–100.0)		40.0 (25.0–55.0)	
Yes/Occasionally	50.0 (32.5–75.0)		45.0 (30.0–65.0)	
Diet Rich in Sodium		0.096		0.210
No	65.0 (42.5–100.0)		55.0 (30.0–75.0)	
Yes/Occasionally	47.5 (25.0–67.5)		45.0 (30.0–60.0)	
Smoking		0.744		0.243
No	50.0 (32.5–67.5)		40.0 (25.0–60.0)	
Yes	52.5 (22.5–77.5)		50.0 (30.0–65.0)	
Alcohol		0.146		0.071
No	55.0 (32.5–77.5)		42.5 (25.0–60.0)	
Yes/Occasionally	45.0 (22.5–67.5)		50.0 (35.0–70.0)	
Exercise		0.545		0.001
Never	42.5 (25.0–75.0)		35.0 (25.0–50.0)	
1–2 times/week	60.0 (22.5–77.5)		50.0 (40.0–65.0)	
>2 times/week	53.8 (35.0–68.8)		65.0 (47.5–75.0)	

**Table 6:** Effects of patient characteristics on baseline QoL (*n* = 100).

	Physical Functioning	Physical Role	Emotional Role	Energy–Fatigue	Emotional Well-Being	Social Functioning	General Health
	β (95% CI)	β (95% CI)	β (95% CI)	β (95% CI)	β (95% CI)	β (95% CI)	β (95% CI)
Gender (female vs. male)	-	−8.2 (−25.8–9.6)	−4.9 (−27.7–17.9)	−10.1 (−20.8–0.7)	-	−3.2 (−14.8–8.3)	−1.1 (−10.6–8.4)
Age (years) (Ref. Cat.: ≤60)							
61–70	-	-	-	14.8 (2.9–26.5)*	-	-	−3.3 (−14.8–8.2)
>70	-	-	-	9.2 (−4.2–22.6)	-	-	−1.6 (−14.8–11.5)
Family Status (Ref. Cat.: Married/Living Together)							
Single/Divorced	-	-	-	−6.8 (−21.4–7.8)	-	−8.5 (−23.4–6.5)	
Widowed	-	-	-	−6.6 (−17.9–4.7)	-	−8.6 (−21.7–4.5)	
Education Level (Ref. Cat.: Primary)							
Secondary	10.1 (−7.8–28.1)	9.9 (−7.6–27.4)	28.5 (5.8–51.3) *	13.2 (3.3–23.2) *	-	6.3 (−5.1–17.8)	17.2 (7.7–26.6) *
University	16.4 (−6.5–39.3)	17.9 (−3.8–39.7)	51.2 (23.4–78.9) *	12.2 (0.4–24.8) *	-	12.8 (0.7–26.4) *	20.9 (8.9–32.8) *
Occupation (pensioner vs. employee)	−2.7 (−20.3–14.9)	-	5.4 (−17.2–28.0)	−7.5 (−20.2–5.2)	-	−1.9 (−12.8–9.1)	2.1 (−10.3–14.6)
No. of Children (Ref.Cat.: None)							
One	8.9 (−19.9–37.7)	-	-	6.7 (−8.5–21.9)	-	-	
Two	−3.5 (−27.2–20.3)	-	-	−8.1 (−22.3–6.2)	-	-	
More than Two	−12.3 (−42.4–17.8)	-	-	−12.5 (−29.8–4.7)	-	-	
CAD Family History (Yes vs. No)	-	-	-	-	−7.8 (−14.8–0.7) *	-	
Other Disease (Yes vs. No)	-	-	-	−7.4 (−16.7–1.9)	-	-	−5.1 (−14.1–3.9)
Smoking (Yes vs. No)	-	-	20.3 (1.9–38.8) *	-	-	-	
Exercise (Ref. Cat.: Never)							
1–2 times/week	7.9 (−10.1–25.9)	−1.5 (−19.7–16.6)	-	3.6 (−5.9–13.0)	6.2 (−1.4–13.8)	-	7.9 (−1.2–16.9)
>2 times/week	34.6 (13.2–56.0) *	19.2 (2.4–40.8) *	-	16.4 (5.7–27.1) *	14.8 (5.7–23.7) *	-	14.7 (4.5–24.8) *
				-	-		

* statistically significant *p*-value.

## Data Availability

Not applicable.
